# Identification of *DELLA* Genes and Key Stage for GA Sensitivity in Bolting and Flowering of Flowering Chinese Cabbage

**DOI:** 10.3390/ijms222212092

**Published:** 2021-11-09

**Authors:** Hongling Guan, Xinmin Huang, Yunna Zhu, Baoxing Xie, Houcheng Liu, Shiwei Song, Yanwei Hao, Riyuan Chen

**Affiliations:** 1 College of Horticulture, South China Agricultural University, Guangzhou 510642, China; hlguan@stu.scau.edu.cn (H.G.); hxmscau@163.com (X.H.); zhuyn326@126.com (Y.Z.); 514027601@stu.scau.edu.cn (B.X.); liuhch@scau.edu.cn (H.L.); swsong@scau.edu.cn (S.S.); 2 College of Biology and Food Engineering, Guangdong University of Petrochemical Technology, Maoming 525000, China; 3 Henry Fok College of Biology and Agriculture, Shaoguan University, Shaoguan 512005, China; 4 College of Life Sciences, South China Agricultural University, Guangzhou 510642, China

**Keywords:** flowering Chinese cabbage, DELLA, gibberellin signaling, bolting and flowering, flower bud differentiation

## Abstract

Flowering Chinese cabbage (*Brassica campestris* L. ssp. *chinensis* var. *utilis* Tsen et Lee) is an important and extensively cultivated vegetable in south China, and its stalk development is mainly regulated by gibberellin (GA). DELLA proteins negatively regulate GA signal transduction and may play an important role in determining bolting and flowering. Nevertheless, no systematic study of the *DELLA* gene family has been undertaken in flowering Chinese cabbage. In the present study, we found that the two-true-leaf spraying of gibberellin A3 (GA_3_) did not promote bolting but did promote flowering, whereas the three-true-leaf spraying of GA_3_ promoted both bolting and flowering. In addition, we identified five *DELLA* genes in flowering Chinese cabbage. All five proteins contained DELLA, VHYNP, VHIID, and SAW conserved domains. Protein-protein interaction results showed that in the presence of GA_3_, all five DELLA proteins interacted with BcGID1b (GA-INSENSITIVE DWARF 1b) but not with BcGID1a (GA-INSENSITIVE DWARF 1a) or BcGID1c (GA-INSENSITIVE DWARF 1c). Their expression analysis showed that the *DELLA* genes exhibited tissue-specific expression, and their reversible expression profiles responded to exogenous GA_3_ depending on the treatment stage. We also found that the *DELLA* genes showed distinct expression patterns in the two varieties of flowering Chinese cabbage. *BcRGL1* may play a major role in the early bud differentiation process of different varieties, affecting bolting and flowering. Taken together, these results provide a theoretical basis for further dissecting the DELLA regulatory mechanism in the bolting and flowering of flowering Chinese cabbage.

## 1. Introduction

Flowering Chinese cabbage is a subspecies of Chinese cabbage that originated in south China. It is now planted throughout the country owing to the increasing consumer demand [1]. The major food product of flowering Chinese cabbage is the stalk, the development of which is directly related to plant quality and yield [2]. Stem elongation, thickening, and flowering are key characteristics of flowering Chinese cabbage stem development. Factors that affect the timing of the bolting and flowering of flowering Chinese cabbage include temperature and plant hormones [3,4,5]. Low temperature and exogenous gibberellin A3 (GA_3_) treatment advance bolting time, stem elongation, and flowering time. Moreover, they display positive additive effects. Low temperature accelerates bolting and flowering by inducing gibberellin (GA) content in the shoot apices; therefore, GA is the main regulator of flowering and bolting in flowering Chinese cabbage.

The regulatory function of GA in plant growth and development is mainly achieved through the GA signaling pathway. DELLA proteins act as the negative regulators of GA signal transduction [6]. GA-induced DELLA degradation is a central regulatory system in the GA signaling pathway [7,8,9]. DELLA proteins contain highly conserved DELLA and TVHYNP domains at the N-terminus, which are important for GA signal perception domains [10,11]. The middle region harbors a nuclear localization signal domain, with a conserved amino acid domain VHIID, leucine repeats LZ, and ploy S/T/V (serine, threonine, and valine). The C-terminus has a conserved GRAS domain, which is a functional structural domain of DELLA proteins and plays a role in regulating DELLA protein activity. VHIID and SAW in the GRAS domain are blocking structural domains.

The *Arabidopsis* genome has five DELLA subfamily members: GA insensitive (GAI), repressor of gal-3 (RGA), RGA-like 1 (RGL1), RGL2, and RGL3 [12,13]. These DELLA proteins belong to the plant-specific GRAS regulatory protein gene family [6,11]. GAI and RGA are crucial for the regulation of plant stem elongation growth in response to GA [13,14,15] and have a functional overlap in inhibiting plant elongation [16,17]. RGL1 and RGL2 play important roles in controlling flower bud differentiation and flower development, respectively [16,17,18,19]. RGL3 acts as a positive regulator in the defense response but performs a minor function in plant development processes [20,21]. These five DELLA proteins in *Arabidopsis* have both redundant and specific functions [21,22]. There are many studies on DELLA proteins in *Arabidopsis*; however, research on flowering Chinese cabbage is still lacking. Advances in research on GA signal transduction pathways in model plants, such as the molecular mechanism of DELLA proteins blocking plant growth and development and the model of GA depression, provide an important basis for studying the mechanism of DELLA proteins in flowering Chinese cabbage.

To understand the role of *DELLA* genes in the bolting and flowering of flowering Chinese cabbage, we isolated five *DELLA* family genes from flowering Chinese cabbage. We then analyzed their expression patterns in different flowering Chinese cabbage tissues and their response to GA_3_ and cold treatment as well as their expression levels in two different cultivars of flowering Chinese cabbage. Finally, we investigated the interaction between DELLA proteins and the GA receptor BcGID1a/b/c using yeast two-hybrid. These data laid the foundation for the further study of DELLA protein function in flowering Chinese cabbage.

## 2. Results

### 2.1. Identification of Key Stage for GA_3_ Sensitivity in Bolting and Flowering of Flowering Chinese Cabbage ‘youlv501’

**Figure 1:**
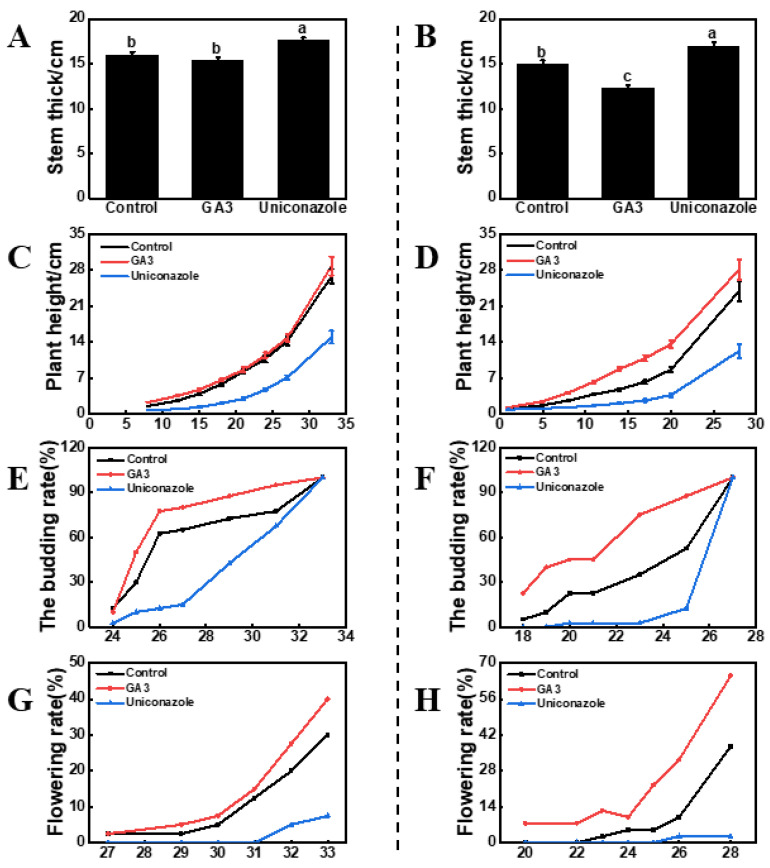
Effect of gibberellin A3 (GA_3_) and uniconazole spraying on the growth of flowering Chinese cabbage. (**A**,**B**) are at 33 and 28 days after treatment, respectively, which is also the harvesting period, and we measured the stem thickness. (**A**,**C**,**E**,**G**): two-true-leaf treatment, (**B**,**D**,**F**,**H**): three-true-leaf treatment. The units on the lower axis are the number of days after treatment in (**C**–**F**). The data represent an average of three replicates ± standard error. Values followed by the same letter are not significantly different using Duncan’s test at *p* < 0.05.

To identify the key stage of stalk development in flowering Chinese cabbage in response to GA_3_ treatments, we treated the plants at the two-true-leaf and three-true-leaf stages with GA_3_ and its inhibitor (uniconazole) separately. To check the effect of these treatments on plant bolting and flowering, we calculated stem diameter, plant height, budding rate, and flowering rate. As shown in Figure 1, there was a different effect on the stem diameters between the treated stages, and the three-true-leaf stage appeared to be more sensitive to GA_3_ treatment. After the two-true-leaf stage treatment, there was no significant difference in stem diameter between the GA_3_-treated and non-treated plants, whereas for the three-true leaf stage, the stem diameter of the GA_3_-treated plants was thinner than that of the control. Similar results were observed for plant height. After the two-true-leaf stage treatment, there was no significant difference in plant height between the GA_3_-treated and non-treated plants, whereas after the three-true-leaf stage treatment, the plant height of the GA_3_-treated plants was higher than that of the control. GA_3_ treatment had the same effect on the bolting and flowering of flowering Chinese cabbage, accelerating both bolting and flowering time regardless of the treatment stage (Figure 1). The three-true-leaf stage promoted bolting and flowering more significantly than the two-true-leaf stage. Regardless of the stage, the stem diameter was significantly increased, the plant height was obviously shorter, and the bolting and flowering rates were significantly delayed after uniconazole treatment. Overall, the three-true-leaf stage may be the key stage for stalk development.

### 2.2. Identification and Expression of DELLA Genes in Flowering Chinese Cabbage ‘youlv501’

**Figure 2:**
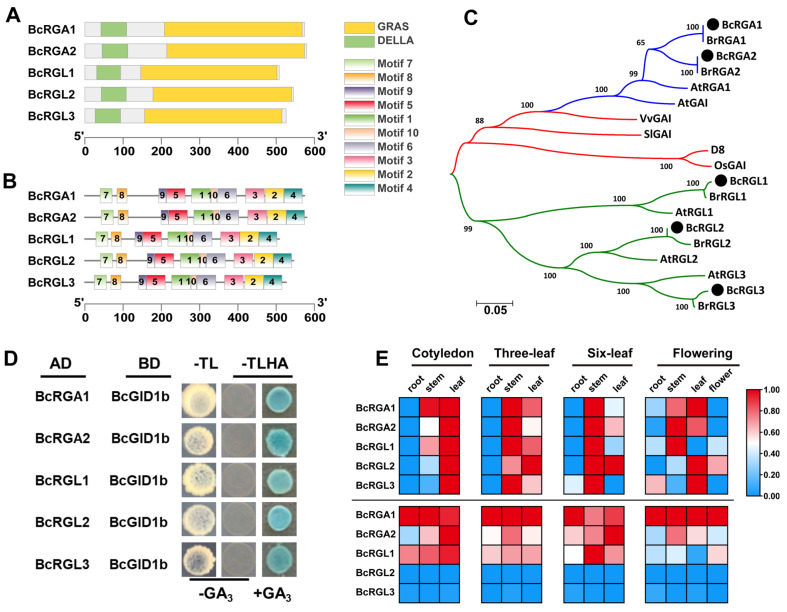
Characterization of DELLA proteins in flowering Chinese cabbage. (**A**) Conserved domain of the *DELLA* genes. Green and yellow represent the DELLA and GRAS domain, respectively. (**B**) Conserved motifs of the *DELLA* genes. Different motifs are represented by different colored boxes with numbers 1−10. (**C**) Phylogenetic tree of DELLA proteins in flowering Chinese cabbage and other species, as determined by the neighbor-joining method. The protein IDs used in the phylogenetic tree are AtRGA1 (NP_178266.1), AtGAI (NP_172945.1), AtRGL1 (NP_176809.1), AtRGL2 (NP_186995.1), AtRGL3 (NP_197251.1), BrRGA1 (XP_009101333.1), BrRGA2 (XP_009114228.2), BrRGL1 (XP_009127484.1), BrRGL2 (XP_009130367.1), BrRGL3 (XP_009107522.1), SlGAI (NP_001234365.1), D8 (NP_001354393.1), OsGAI (XP_015631543.1), and VvGAI (XP_002284648.1). (**D**) Detection of interactions among DELLA proteins and BcGID1b using Y2H. Yeast cells with different construct combinations were grown on selective media without Trp and Leu (-TL) and were tested for interactions on selective media without Trp, Leu, His, and Ade (−TLHA). (**E**) Expression profiles of flowering Chinese cabbage *DELLA* genes in various organs. Above the line is a horizontal comparison, with each column exhibiting normalization independently, and below the line is a vertical comparison, with each row displaying normalization independently. GA_3_, gibberellin A3.

As GA regulates plant growth and development mainly through the GA signaling pathway, in which DELLA proteins act as key regulators, we characterized the DELLA proteins of flowering Chinese cabbage. An in silico search was performed in the Brassica Database (http://brassicadb.cn/#/BLAST/, accessed on 20 August 2021) using *Arabidopsis* DELLA protein RGA sequences as queries for BLAST searches. Five genes were predicted to encode the putative DELLA proteins. The full-length cDNA of the five *DELLA* genes was further confirmed by reverse transcription polymerase chain reaction (RT-PCR) amplification, indicating that the corresponding coding sequences ranged from 1524 to 1740 bp (Table 1, Figure S1 and Table S1). We used Expasy and Wolf Psort online software to analyze the physicochemical properties of the predicted proteins based on the sequence results. The physicochemical properties are shown in Table 1. All DELLA contained conserved DELLA and GRAS (Figure 2A). Multiple sequence alignment revealed that they also contained the DELLA, VHYNP, VHIID, and SAW domains (Figure S2). Motif searches in the MEME online program indicated that all DELLA contained the same conserved motif numbers (Table S2). Motif 7 and 8 represent the DELLA domain, whereas motifs 1, 2, 3, 4, 5, 6, 9, and 10 are in the GRAS domain (Figure 2B).

To adopt a nomenclature consistent with that of *Arabidopsis* DELLA proteins, we conducted phylogenetic analyses on different DELLA proteins from different plant sequenced genomes comprising Chinese cabbage, *Arabidopsis thaliana*, tomato, grape, rice, and maize sequences. The phylogenetic trees showed that the DELLA proteins in flowering Chinese cabbage had the closest relationship with Chinese cabbage, followed by *Arabidopsis*. The DELLA proteins in flowering Chinese cabbage were named according to their phylogenetic trees (Figure 2C). The evolutionary tree also showed that BcRGA1 and BcRGA2 are more closely related, whereas BcRGL1, BcRGL2, and BcRGL3 belong to one branch and are more closely related. Using the *Arabidopsis* protein database as a reference, online software STRING was used to predict the potential interaction proteins of DELLA family members. The results showed that BcRGA1, BcRGA2, BcRGL1, BcRGL2, and BcRGL3 interacted with the GID1A, GID1B, GID1C, PIF3, and SLY1 proteins (Figure S1B). To verify the interaction between the DELLA proteins and GA receptor GID1 in flowering Chinese cabbage, we performed yeast two-hybrid experiments in the presence or absence of GA_3_. The yeast double-hybrid result showed that the five DELLA proteins could only interact with BcGID1b in the presence of GA_3_, and none of the five DELLA proteins interacted with the GA receptors, BcGID1a and BcGID1c, either with or without GA_3_ (Figure 2D and Figure S1C,D). The expression patterns of the *BcRGA1*, *BcRGA2*, *BcRGL1*, *BcRGL2*, and *BcRGL3* genes were examined in different tissues of flowering Chinese cabbage, including roots, stems, leaves, and flowers. The relative expression was calculated using the 2^−ΔΔCt^ method. The results showed that all the *DELLA* genes were expressed in all the evaluated tissues. The expression profiles of these genes in the cotyledon, three-true-leaf, six-true-leaf, and flowering stages were similar, with high expression levels in the stems and leaves and low expression levels in the roots and flowers (Figure 2E and Figure S3). The expression levels of *BcRGA1*, *BcRGA2,* and *BcRGL1* were higher than those of the other three genes (Figure 2E and Figure S3).

### 2.3. DELLA Genes Responded Differentially to GA_3_ between Two-True-Leaf Stage and Three-True-Leaf Stage Treatments

**Figure 3:**
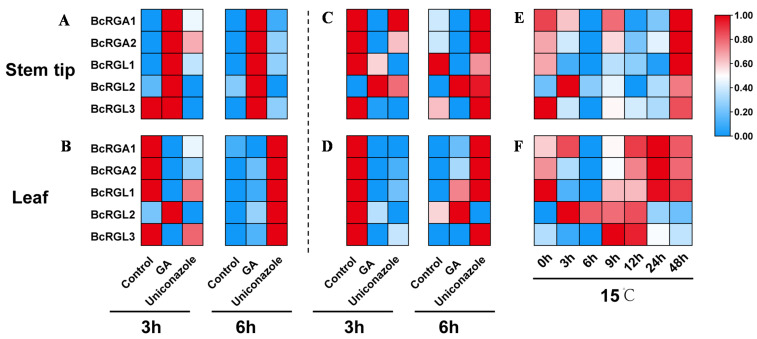
Expression profiles of flowering Chinese cabbage *DELLA* genes under gibberellin A3 (GA_3_), uniconazole, and 15 °C treatments. (**A**,**B**): two-true-leaf treatment, (**C**–**F**): three-true-leaf treatment. Each column displays normalization independently.

To determine whether the DELLA proteins respond to GA differentially between the two- and three-true-leaf stages, we examined the expression level of *DELLA* genes at these two stages after GA_3_ and uniconazole (GA inhibitor) treatment using RT-PCR-based expression analysis. The relative expression was calculated using the expression levels of the housekeeping gene Actin2/GAPDH and the 2^−ΔΔCt^ method. The trend of *DELLA* genes expression in leaves was the same for both the two- and three-true-leaf treatments. The expression of *DELLA* genes was downregulated after 3 h of GA_3_ treatment and upregulated after 6 h of uniconazole treatment. In the stem tip, the expression pattern of the *DELLA* genes was different between the two- and three-true-leaf stage treatments. After two-true-leaf GA_3_ treatment, the expression of *DELLA* genes was upregulated, whereas the expression of the other *DELLA* genes, except *BcRGL2*, was downregulated after the three-true-leaf GA_3_ treatment (Figure 3, Figures S4 and S5). 

Low temperature promotes bolting and flowering of flowering Chinese cabbage ‘youlv501’ by inducing GA content. To evaluate whether temperature affects the *DELLA* genes, we treated flowering Chinese cabbage at 15 °C in the three-true-leaf stage. Most *DELLA* genes exhibited inhibition after 3 h of treatment, and the most significant downregulation in the expression was observed after 6 h of treatment. However, the expression of *BcRGL2* was upregulated by the treatment (Figure 3E,F and Figure S6). Low temperature downregulated the expression of most of the *DELLA* genes, which was consistent with the spraying of GA_3_ in the three-true-leaf stage; this indicated that the three-true-leaf stage is the key stage for bolting and flowering, and *DELLA* genes play an important role in this process.

### 2.4. Bolting and Flowering-Related Genes Responded Differentially to GA between Two- and Three-True-Leaf Stage Treatments

**Figure 4:**
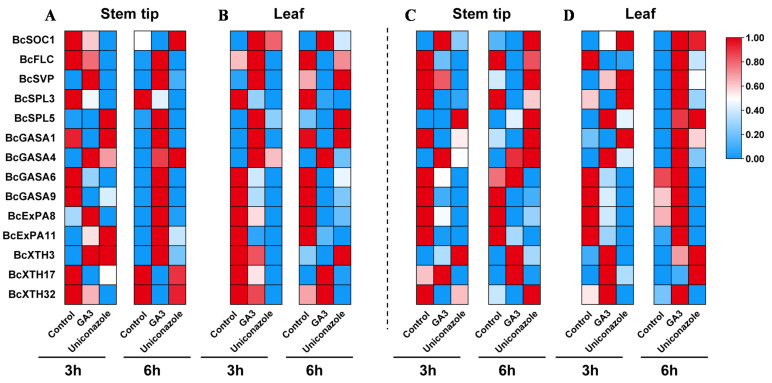
Expression profiles of flowering and elongation genes in flowering Chinese cabbage. (**A**,**B**): two-true-leaf treatment, (**C**,**D**): three-true-leaf treatment. Each column displays normalization independently. GA3, gibberellin A3.

In combination with a previous study [2], we selected five flowering-related genes, *SOC1*, *FLC*, *SVP*, *SPL3*, and *SPL5*; five cell elongation-related genes, *EXP8*, *EXP11*, *XTH3*, *XTH17*, and *XTH32*; and four GA-regulated proteins, *GASA1*, -*4*, -*6*, and -*9*, to analyze the expression characteristics of these genes after GA_3_ treatment (Figure 4, Figures S7 and S8). *SOC1* is a flowering promoter, and *FLC* and *SVP* are flowering repressors [23,24,25,26]. The expression of *SOC1* was increased in the leaves and stem tips after GA_3_ treatment, except in the stem tips of the two-true-leaf stage. SPL is an interactive DELLA protein [27]. The expression of *SPL3* and *SPL5* was significantly different. After GA_3_ treatment, the expression of *SPL3* was downregulated, whereas the expression of *SPL5* was either upregulated or unchanged. The expression of GA-regulated proteins also differed, with GA_3_ treatment upregulating the expression of *GASA4* and downregulating the expression of *GASA1*, -*6*, and *-9*. Expansin proteins (ExPA) and xyloglucan endotransferases (XTH) are key factors in cell elongation. The expression of *XTH3* and *XTH17* was increased by GA_3_ treatment, especially in the three-true-leaf stage.

Concomitantly, we analyzed the correlation of these genes with the *DELLA* genes in leaves and stem tips during different treatments with GA_3_ (Figure S9). The correlations between genes varied at different stages due to changes in gene expression. The correlation between genes was not significant in the two-true-leaf stage stem tips. In the three-true-leaf stage stem tips, *BcRGA1* was significantly and negatively correlated with *BcXTH17*. In the two-true-leaf stage leaves, all the *DELLA* genes were positively correlated with *BcSPL5* and *BcXTH3*. In the three-true-leaf stage leaves, *BcRGL2* was significantly and positively correlated with *BcGASA6*, -*9*, *BcExPA8*, -*11*, and *BcXTH32*.

### 2.5. Five DELLA Genes Showed Distinct Expression Patterns in Two Varieties of Flowering Chinese Cabbage

**Figure 5:**
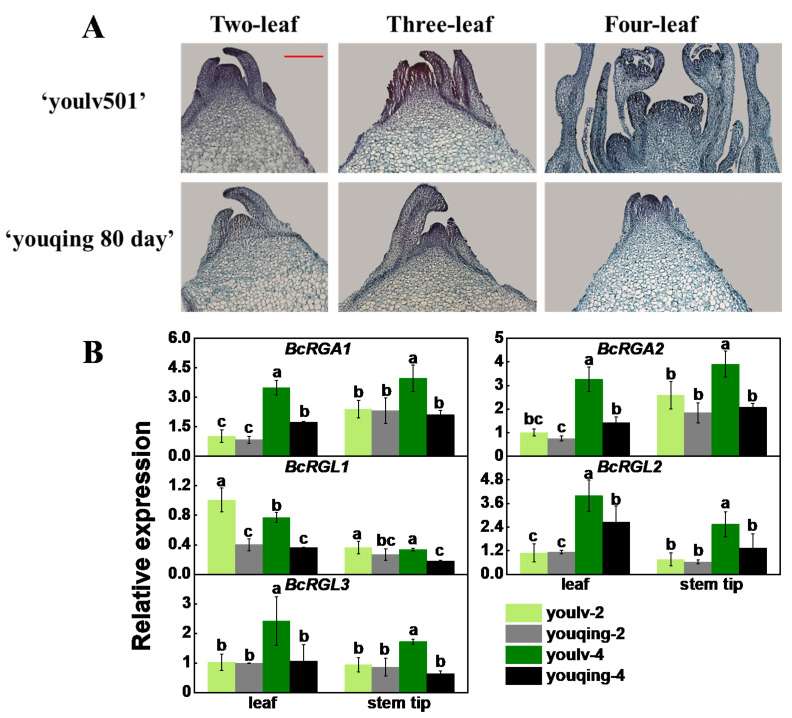
Expression analysis of *DELLA* genes in the ‘youlv501’ and ‘youqing 80 day’ varieties of flowering Chinese cabbage. (**A**) Stem tip longitudinal structures of ‘youlv501’ and ‘youqing 80 day’ flowering Chinese Cabbage. All the pictures were taken under a 10 × microscope, and the scale was unified at 200 μm. (**B**) Among them, youlv-2 and youqing-2 represent the two-true-leaf stage of ‘youlv501’ and ‘youqing 80 day’ respectively. youlv-4, and youqing-4 represent the four-true-leaf stage of ‘youlv501’ and ‘youqing 80 day’, respectively. The data represents an average of three replicates ± standard error. Values followed by the same letter are not significantly different using Duncan’s test at *p* < 0.05.

An inappropriate sowing season may cause abnormal bolting and flowering of flowering Chinese cabbage; therefore, various varieties suited to different seasons have been developed. ‘youlv501’ and ‘youqing 80 day’ are two varieties of flowering Chinese cabbage, which are an early-maturing variety and a late-maturing variety, respectively. Using paraffin sections, we observed the stem tip structure of two varieties of flowering Chinese cabbage. From the horizontal section of the stem tips, we found that ‘youlv501’ has larger pith cells than ‘youqing 80 day’ at the three- and four-true-leaf stages (Figure S10). As illustrated in the longitudinal section of the stem tips, ‘youlv501′ developed faster than ‘youqing 80 day’ at all three stages, especially during the four-true-leaf stage. In the two-true-leaf stage, both varieties did not complete the flower bud differentiation; however, in the four-true-leaf stage, the flower bud differentiation of ‘youlv501’ was completed (Figure 5A). The expression levels of all the *DELLA* genes were significantly different at the four-true-leaf stage, whereas only the expression level of *BcRGL1* was significantly different at the two-true-leaf stage (Figure 5B), indicating its potential role in the bolting and flowering of flowering Chinese cabbage in these cultivar variations.

## 3. Discussion

We identified five *DELLA* genes (*BcRGA1*, *BcRGA2*, *BcRGL1*, *BcRGL2*, and *BcRGL3*) in flowering Chinese cabbage, which are members of the plant-specific GRAS family. All five *DELLA* contain the DELLA, TVHYNP, VHIID, and SAW domains [10,11,19]. These domains have been implicated in the perception of GA and the subsequent destabilization of DELLA proteins [16,28,29,30]. Mutations to either of these domains can result in a semi-dominant, GA-insensitive, and dwarfed phenotype in various plant species [15,31,32]. *Arabidopsis* and Chinese cabbage have five members of the DELLA subfamily, whereas monocots, such as rice and barley, have only one [15,19,33]. The names of the individual DELLA introduced here are intended to imply functional homology to specific *Arabidopsis* DELLA proteins.

The interaction between GID1 and DELLA proteins in rice and plum is dependent on GA [34,35]. In addition, GID1 interacts with DELLA proteins even in the absence of GA, and the presence of GA enhances the interaction between GID1 and DELLA proteins [36]. Therefore, GA-dependent and -independent pathways are present in plants [37]. In our study, the DELLA proteins only interacted with the GA receptor BcGID1b in the presence of GA_3_, probably because BcGID1b has a greater binding ability to GA than BcGID1a and BcGID1c [38]. Gene knockouts, overexpression of individual *DELLA* genes, and further characterization of the DELLA proteins will help elucidate the role of these proteins in the development of flowering Chinese cabbage.

The transcript levels of four *DELLA* genes in cucumber have been analyzed in different tissues and organs, including roots, stems, leaves, male flower buds, female flower buds, and fruits, and all four genes are expressed in the examined tissues, with the highest expression in stems and male flower buds [39]. In *Arabidopsis*, five *DELLA* genes are expressed in different tissues, and the expression of *RGA1*, *RGA2*, and *RGL1* is higher than that of *RGL2* and *RGL3*, similar to the expression profiles of the *DELLA* genes in flowering Chinese cabbage [40]. We analyzed the expression of five *DELLA* genes in the roots, stems, leaves, and flowers of flowering Chinese cabbage found that the *DELLA* genes were mainly expressed in the stem tips and leaves, and the highly expressed genes were consistent with those in *Arabidopsis*. It is noteworthy that during the cotyledon stage, expression was mainly present in the leaves, whereas during the three- and six-true-leaf stages, the expression was higher in the stems than in the leaves. It is possible that *DELLA* genes act mainly in the stem tip as flowering Chinese cabbage enters the bud differentiation and rapid growth phase.

GA promotes bolting and flowering in mustard, radish, and Chinese cabbage, but the results vary among varieties, different stages, and different environmental conditions [41,42,43,44]. Uniconazole (a GA biosynthesis inhibitor) can inhibit bolting and flowering and improve yield in purple kale and cauliflower [41]. In flowering Chinese cabbage, two-true-leaf spraying of GA_3_ did not promote bolting but did promote flowering, whereas three-true-leaf spraying of GA_3_ promoted bolting and flowering. Uniconazole significantly inhibited bolting and flowering in the two- and three-true-leaf treatments. This indicates that GA_3_ can affect bolting and flowering, but the effect of GA_3_ depends on the treatment stage. The experiment was conducted with an early maturing variety, ‘youlv501′, which may have initiated floral bud differentiation earlier. In the two-true-leaf stage, flowering Chinese cabbage mainly undergoes floral bud differentiation, and the main effect of GA may affect floral bud differentiation, thus affecting plant flowering. In contrast, during the three-true-leaf stage, in addition to floral bud differentiation, the bolting signal is initiated; thus, GA may also affect stem elongation and flowering. At the three-true-leaf stage, the expression of the *DELLA* genes, except *BcRGL2,* was downregulated after 3 h of GA treatment. This indicates that *BcRGA1*, *BcRGA2*, *BcRGL1*, and *BcRGL3* may inhibit stem growth and development, whereas GA_3_ treatment, which degrades them, initiates stem elongation and development. These findings are consistent with the current understanding of DELLA proteins as GA-responsive repressors of plant growth [17]. *RGL2* is a negative regulator of GA responses that acts specifically to control seed germination rather than stem elongation [19]. We found that *BcRGL2* responded differently to GA than the other four genes, suggesting that *BcRGL2* may have a different function in flowering Chinese cabbage.

GA affects flowering Chinese cabbage bolting and flowering through DELLA proteins, but the exact molecular mechanism is not clear. We searched for the relationship between *DELLA* genes, cell elongation, and flower-related genes by detecting their expression and correlation. In the three-true-leaf stage stem tips, GA_3_ treatment caused the downregulation of the expression of *DELLA* genes, increased the expression of flowering-promoting factor *BcSOC1*, and decreased the expression of flowering-inhibiting factors *BcFLC* and *BcSVP*. Furthermore, GA_3_ treatment upregulated the expression of *BcGASA4*, *BcXTH3*, and *BcXTH17*. Previous studies have shown that DELLA proteins interact with FLC and SPL to repress the flowering transition [23,27]. GA induces the expression of *EXPA* and *XTH* genes through signal transduction pathways to promote cell wall relaxation and thus cell elongation [45,46]. Overexpression of *AtGASA6* promotes cell elongation [47]. Therefore, we hypothesized that GA_3_ spraying causes the degradation of *DELLA*, *BcFLC,* and *BcSPL3* and upregulates *BcSOC1* expression, thus promoting flowering in flowering Chinese cabbage. Furthermore, GA_3_ spraying upregulated *BcGASA4* expression and promoted *BcXTH3* and *BcXTH17* gene expression, which promotes cell wall relaxation and ultimately the elongation of flowering Chinese cabbage stems. Based on the Pearson correlation, *BcRGA1* was significantly and negatively correlated with *BcXTH17*. It is possible that *BcRGA1* and *BcXTH17* play a key role in the stem growth of flowering Chinese cabbage, and follow-up studies should be conducted to verify this role.

In general, flower bud differentiation starts in the two- and three-true-leaf stages, and the flower bud differentiation stage varies among different varieties [48]. The early and late development of flower buds is different for the three varieties with different maturities, and they all start flower bud differentiation in the two- or three-true-leaf stages, but the development speed is generally early maturity < medium maturity < late maturity. ‘youlv501’ and ‘youqing 80 day’ are the two varieties of flowering Chinese cabbage, representing an early-maturing variety and a late-maturing variety, respectively. To investigate the relationship between *DELLA* genes and bud differentiation, we used the early-maturing variety ‘youlv501’ and the late-maturing variety ‘youqing 80 day’ for the experiment. Paraffin section results showed that ‘youlv501’ developed faster than ‘youqing 80 day’ at all stages. The quantitative results showed that *BcRGL1* was significantly expressed at the two-true-leaf stage. *RGL1* appears to be a more diverse negative regulator in *Arabidopsis* and is associated with seed germination, stem elongation, leaf expansion, and flower development [49]. Nevertheless, *BcRGL1* may play a major role in the early bud differentiation process of different varieties, affecting plant bolting and flowering in flowering Chinese cabbage.

## 4. Materials and Methods

### 4.1. Cloning of DELLA Genes in Flowering Chinese Cabbage

As *Arabidopsis DELLA* genes have been reported previously [12], all the protein sequences from these two gene families were extracted from the Arabidopsis Information Resource database (https://www.arabidopsis.org/, accessed on 10 August 2021) and used as queries for a BLASTP search, with an e-value threshold of <1 in the Brassica Database (http://brassicadb.cn/#/BLAST/, accessed on 10 August 2021). Five *DELLA* genes were found in Brassica cabbage, and primers were designed based on their sequences (Table S3). 

### 4.2. Phylogenetic and Sequence Analysis of DELLA Genes in Flowering Chinese Cabbage

The *DELLA* sequences of some other species and flowering Chinese cabbage were subjected to multiple sequence alignment. Multiple sequence alignment was conducted using Muscle with default parameters, and the results were used to construct a neighbor-joining phylogenetic tree in MEGA7.0, with the bootstrap value set to 1000 [50].

Conserved motifs were analyzed using the Multiple Em for Motif Elicitation (MEME) online program (http://meme-suite.org, accessed on 15 August 2021) with the following parameters: the number of repetitions was set to zero or one, and the maximum number of motifs was 10 [51]. The conserved domain of *DELLA* was analyzed using the NCBI Batch CDD online program (https://www.ncbi.nlm.nih.gov/Structrue/bwrpsb/bwrpsb.cgi, accessed on 15 August 2021). Conserved motifs and gene domains were visualized using TBtools software [52]. pI/MW was computed using the Expasy online program (https://web.expasy.org/compute_pi/, accessed on 15 August 2021) [53]. Protein subcellular localization was predicted using Wolf Psort (https://wolfpsort.hgc.jp/, accessed on 15 August 2021) [54].

### 4.3. Interaction Analysis of DELLA Proteins

Protein interaction analysis prediction for DELLA proteins was completed using the website STRING 11.5 (https://string-db.org/, accessed on 20 August 2021) [55]. PPI maps were visualized using Cytoscape 3.8.2 software [56]. The full-length coding sequences of five *DELLA* and *BcGID1s* genes were amplified from a mixture of stem and leaf cDNA. After amplification (95 °C for 2 min, followed by 35 cycles at 95 °C for 30 s, 58 °C for 30 s, and 72 °C for 1 min), the *DELLA* and *BcGID1s* genes were cloned into the pGAD and pDBD vectors (Clontech, Takara Bio Inc., Shiga, Japan), respectively. The yeast strain AH109 (Clontech) was used for transformation. Diploids were selected on a medium lacking Trp and Leu (TL), and interactions were validated using HIS3 and ADE2 reporter genes in a medium lacking Trp, Leu, His, and Ade (TLHA). Manipulation and analysis of Y2H were performed according to the manufacturer’s instructions in the Clontech Yeast Protocols Handbook, and all experiments were repeated three times independently.

### 4.4. Plant Growth Conditions, GA_3_, and Cold Treatment

Flowering Chinese cabbage plants were planted in pots with perlite and cultivated in the glasshouse of college horticulture at the South China Agriculture University. The growth conditions were as follows: 14 h-day/10 h-night cycles, 25/20 °C day/night temperature, 70–80% relative humidity, and 300 mol m^−2^ s^−1^ intense light.

The ‘youlv501′ flowering Chinese cabbage was used as the experimental material in the treatments with GA and its inhibitor (uniconazole). When the young seedlings were at the two- and three-true-leaf stages, plants were sprayed with GA_3_ (200 mg/L) and uniconazole (10 mg/L). Shoots (including the stem tip and leaf tissue) were collected from both the treated and control plants at 0, 3, and 6 h. In the low-temperature treatment, when the young seedlings were at the three-true-leaf stage, plants were treated at 15 °C, and shoots (including the stem tip and leaf tissue) were collected from both the treated and control plants at 0, 3, 6, 9, 12, 24, and 48 h. ‘Youlv501’ and ‘youqing 80 day’ were used as the experimental material to evaluate different varieties. Shoots (including the stem tip and leaf tissue) were collected when the young seedlings were at the two- and three-true-leaf stages. Three biological replicates were used for each condition, and ten seedlings were used for each biological replicate. All samples were subsequently frozen in liquid nitrogen and stored at −80 °C for RNA extraction.

### 4.5. Histological Analysis

The stem tips (5 mm) of different varieties (‘youlv501’ and ‘youqing 80 day’) were collected and immersed in formaldehyde alcohol acetic acid solution (70% alcohol: acetic acid: formaldehyde = 90:5:5), placed under a vacuum for 20 min, and incubated for 48 h at 4 °C. The samples were then dehydrated in a gradient of ethanol and embedded in paraffin. Subsequently, 8 mm thick sections were stained with reddish-green stain and observed under a microscope.

### 4.6. RNA Isolation and RT-PCR Analysis

Total RNA was isolated from three replicates of each sample using the Promega Plant RNA Kit, which contained a genomic DNA elimination step. Total RNA was quantified spectrophotometrically by measuring the absorbance at 260 nm and the absorbance ratio of 260/280 nm using a Nanodrop (Thermo Fisher Scientific, Waltham, MA, USA) and 2% *w*/*v* agarose gel. First-strand cDNA was reverse transcribed using an Eastep^®^ RT Master Mix kit (Promega Corporation, Madison, WI, USA). Gene-specific primers were designed, and their sequences are listed in Table S3.

To determine the expression levels of the genes, fluorescent quantitative PCR was performed using a LightCycler 480 real-time PCR system (Roche, Basel, Switzerland) with SYBR Premix Ex Taq (Takara Bio Inc., Shiga, Japan), and the relative gene expression was calculated using the expression levels of the housekeeping gene *Actin2*/*GAPDH* and the 2^−ΔΔCt^ method [57]. Gene expression profiles were visualized as a heatmap using TBtools software [52].

## 5. Conclusions

In this study, we isolated five *DELLA* genes from flowering Chinese cabbage, all of which interacted with the GA receptor BcGID1b in the presence of GA_3_. Based on the different morphologies of the two- and three-true-leaf stage GA_3_ treatments on the stem diameters and the reversible expression trend of *DELLA* genes, we believe that the three-true-leaf stage is likely the key stage for bolting regulation. Moreover, *BcRGL1*, which displayed variable expression between the early-maturing variety ‘youlv501’ and the late-maturing variety ‘youqing 80 day’ at the two-true-leaf stage, may be the key factor regulating bolting of flowering Chinese cabbage.

## Figures and Tables

**Table 1:** Physicochemical properties of *DELLA* genes in flowering Chinese cabbage.

Gene	CDS (bp)	AA	pI	MW (KD)	Subcellular Localization
*BcRGA1*	1722	573	4.94	142.7	chlo: 6, nucl: 6, cyto: 1
*BcRGA2*	1740	579	4.93	143.4	nucl: 6.5, nucl_plas: 4, chlo: 3, cyto: 3
*BcRGL1*	1524	507	4.99	126.0	nucl: 6, chlo: 3, cyto: 3, cysk: 2
*BcRGL2*	1635	544	4.94	133.4	chlo: 6, nucl: 3, cyto: 2, mito: 2
*BcRGL3*	1578	525	4.95	129.4	mito: 6, nucl: 3.5, nucl_plas: 3.5, plas: 2.5, chlo: 2

CDS: coding sequence, AA: amino acid, MW: molecular weight.

## Data Availability

Data available in a publicly accessible repository.

## Supplementary Materials

The following are available online at https://www.mdpi.com/article/10.3390/ijms222212092/s1.
