# Supplementary material for: Identification of DELLA Genes and Key Stage for GA Sensitivity in Bolting and Flowering of Flowering Chinese Cabbage
Source: Int J Mol Sci. 2021 Nov 9;22(22):12092. doi: 10.3390/ijms222212092 (PMC8624557; doi:10.3390/ijms222212092)
Supplement: Supplementary file 1 [file ijms-22-12092-s001.zip › Editing Certificate.pdf]

editage

Editing Certificate

This document certifies that the manuscript listed below has been edited to ensure language and grammar accuracy and is error free in these aspects. The edit was performed by professional editors at Editage, a division of Cactus Communications. The author's core research ideas were not altered in any way during the editing process. The quality of the edit has been guaranteed, with the assumption that our suggested changes have been accepted and the text has not been further altered without the knowledge of our editors.

MANUSCRIPT TITLE

Identification of DELLA genes and key stage for GA sensitivity in bolting and flowering of flowering Chinese cabbage

AUTHORS

Hongling Guan1, Xinmin Huang1, 2, Yunna Zhu1, 3, Baoxing Xie1, 4, Houcheng Liu1, Shiwei Song1, Yanwei Hao1*, Ri-yuan Chen1*

ISSUED ON

October 30, 2021

JOB CODE

GSSON_21

THE EDITAGE Promise QUALITY GUARANTEE

Vikas Narang

Vikas Narang
Chief Operating Officer - Editage

editage

Editage, a brand of Cactus Communications, offers professional English language editing and publication support services to authors engaged in over 1300 areas of research. Through its community of experienced editors, which includes doctors, engineers, published scientists, and researchers with peer review experience, Editage has successfully helped authors get published in internationally reputed journals. Authors who work with Editage are guaranteed excellent language quality and timely delivery.

GLOBAL : +1(833) 979-0061 | request@editage.com

CHINA : 400-120-3020 | fabiao@editage.cn

CACTUS

IMPACT SCIENCE impact.science

researcher.life

lifesciences.cactusglobal.com
