# Supplementary material for: Identification of DELLA Genes and Key Stage for GA Sensitivity in Bolting and Flowering of Flowering Chinese Cabbage
Source: Int J Mol Sci. 2021 Nov 9;22(22):12092. doi: 10.3390/ijms222212092 (PMC8624557; doi:10.3390/ijms222212092)
Supplement: Supplementary file 1 [file ijms-22-12092-s001.zip › Supplementary Figure-modified.pdf]

International Journal of
Molecular Sciences

MDPI

Article

Identification of *DELLA* genes and key stage for GA sensitivity in bolting and flowering of flowering Chinese cabbage

Hongling Guan[1], Xinmin Huang[1, 2], Yunna Zhu[1, 3], Baoxing Xie[1, 4], Houcheng Liu[1], Shiwei Song[1], Yanwei Hao[1*], Ri-yuan Chen[1*]

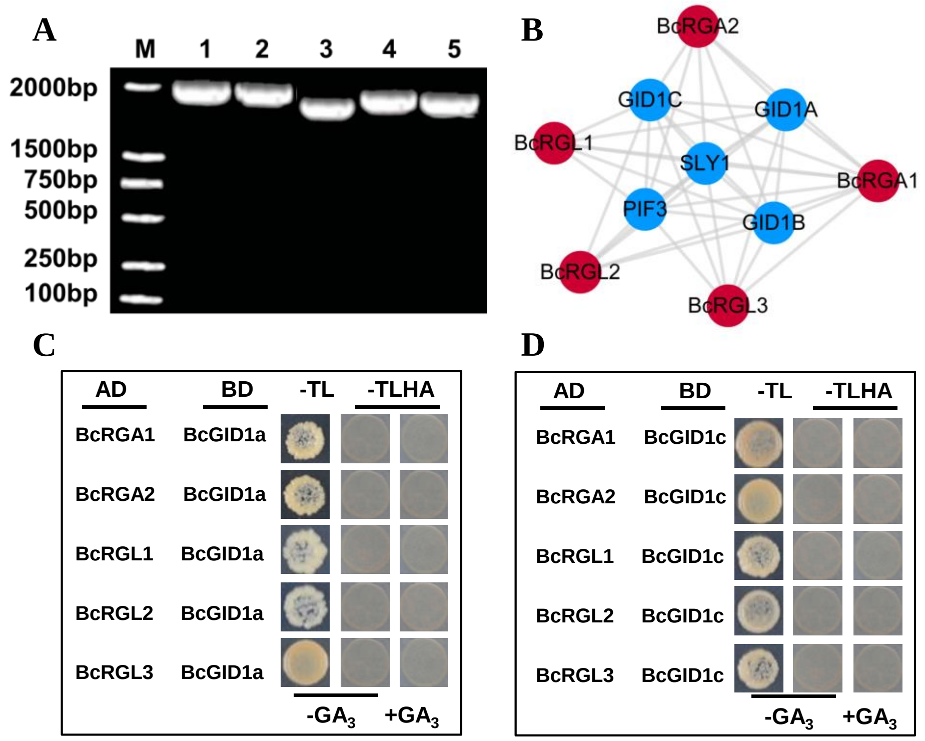

Figure S1. Interaction analysis of DELLA proteins. (**A**) Electrophoresis amplifying the target fragment of flowering Chinese cabbage *DELLA* genes. M: DL2000 marker; 1, 2, 3, 4, and 5 are *BcRGA1*, *BcRGA2*, *BcRGL1*, *BcRGL2*, and *BcRGL3*, respectively. (**B**) *Arabidopsis thaliana* was selected as the model plant and the STRING interactive database was used to construct the interaction network between DELLA and related proteins. (**C**/**D**) Detection of interactions among DELLA proteins and BcGID1a/c using Y2H.

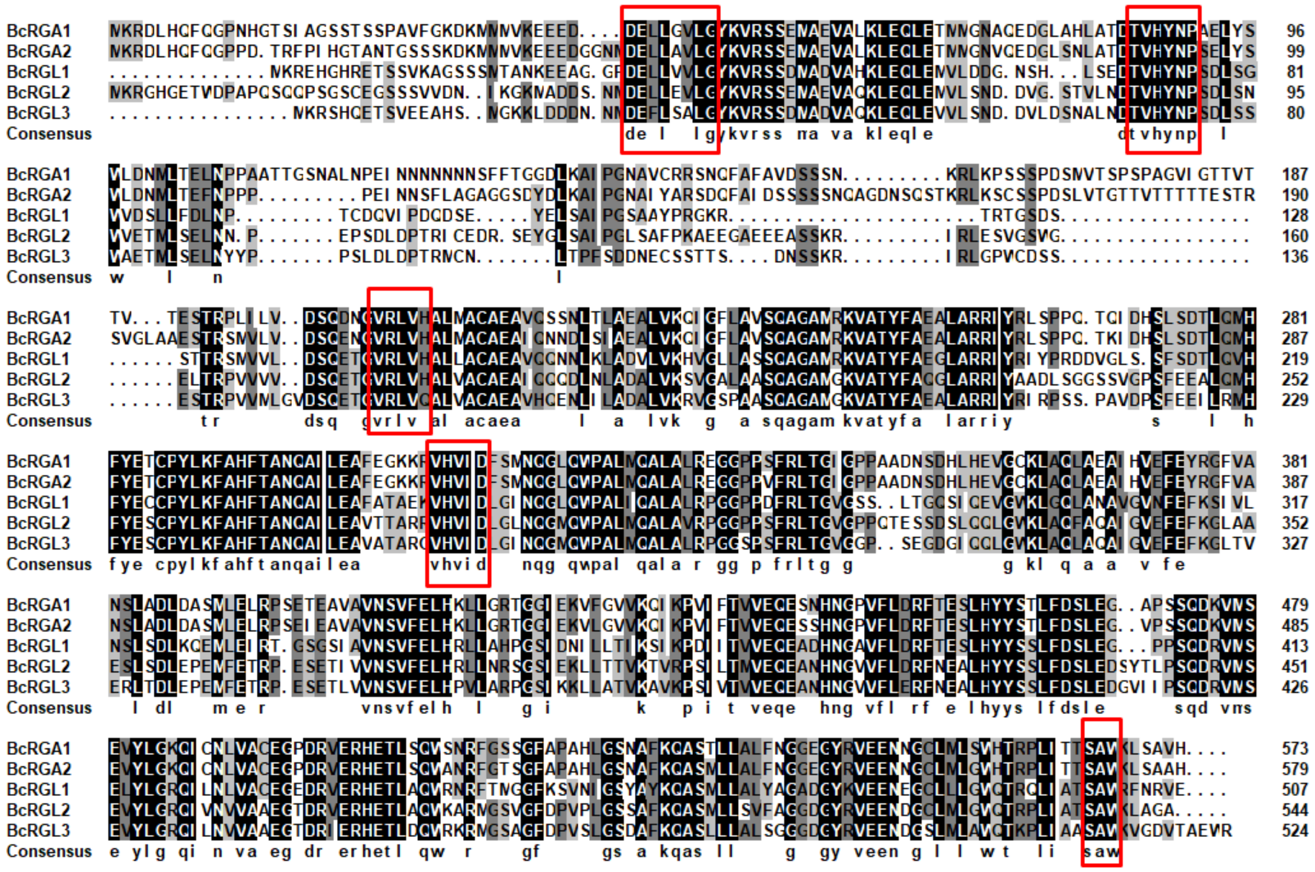

Figure S2. Multiple alignments of amino acid sequence of flowering Chinese cabbage *DELLA* genes. Red boxes represent conserved structural domains.

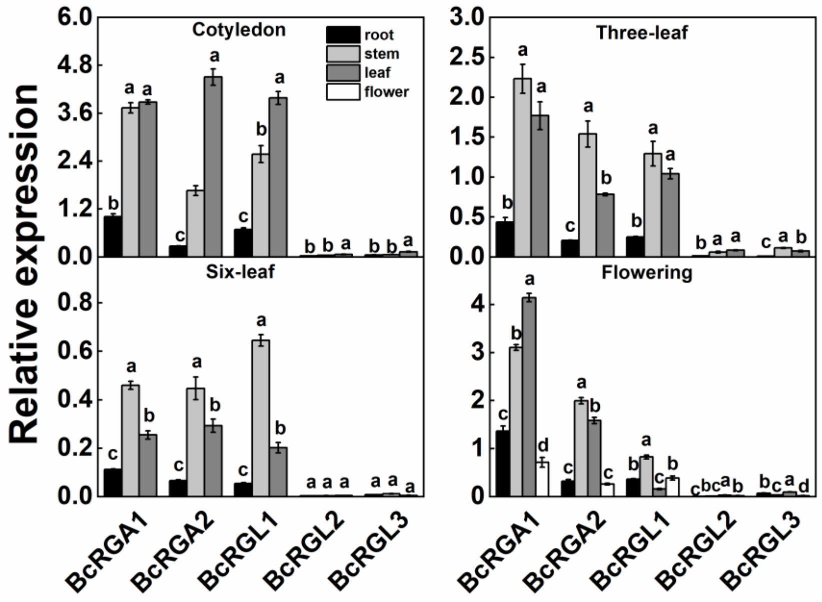

Figure S3. Expression profiles of flowering Chinese cabbage *DELLA* genes in various organs. The data represent an average of three replicates ± standard error. Values followed by the same letter are not significantly different using Duncan's test at $p < 0.05$.

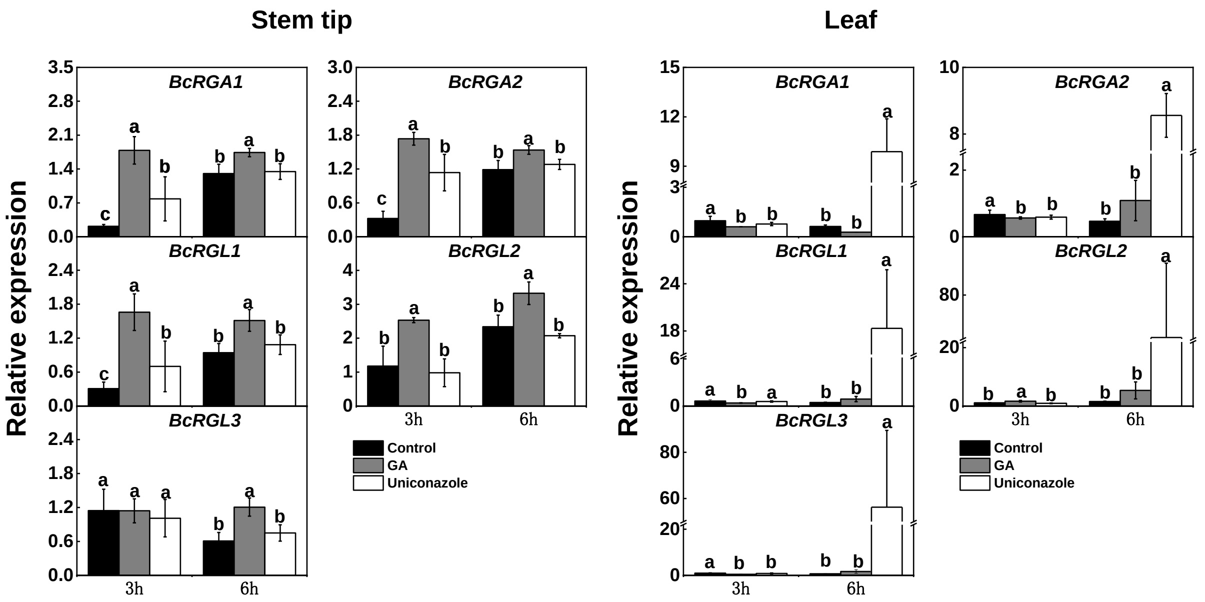

Figure S4. Expression profiles of flowering Chinese cabbage *DELLA* genes under two-true-leaf stage treatments with gibberellin A3 (GA_3) and uniconazole. The data represent an average of three replicates ± standard error. Values followed by the same letter are not significantly different using Duncan's test at $p < 0.05$.

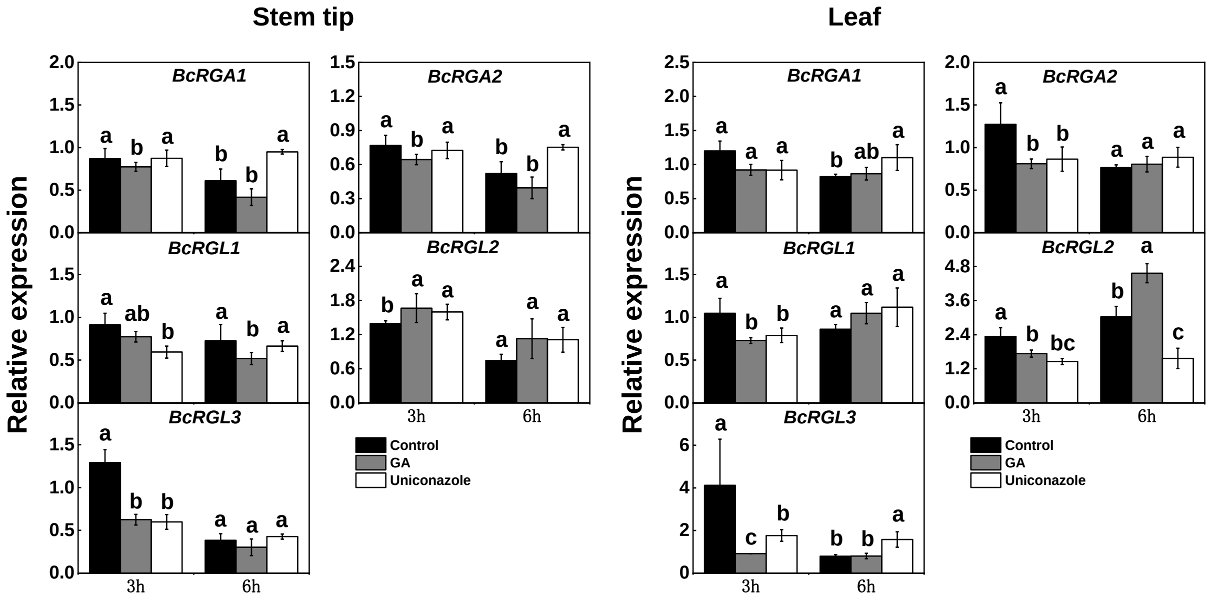

Figure S5. Expression profiles of flowering Chinese cabbage *DELLA* genes under three-true-leaf stage treatments with gibberellin A3 (GA_3) and uniconazole. The data represent an average of three replicates ± standard error. Values followed by the same letter are not significantly different using Duncan's test at $p < 0.05$.

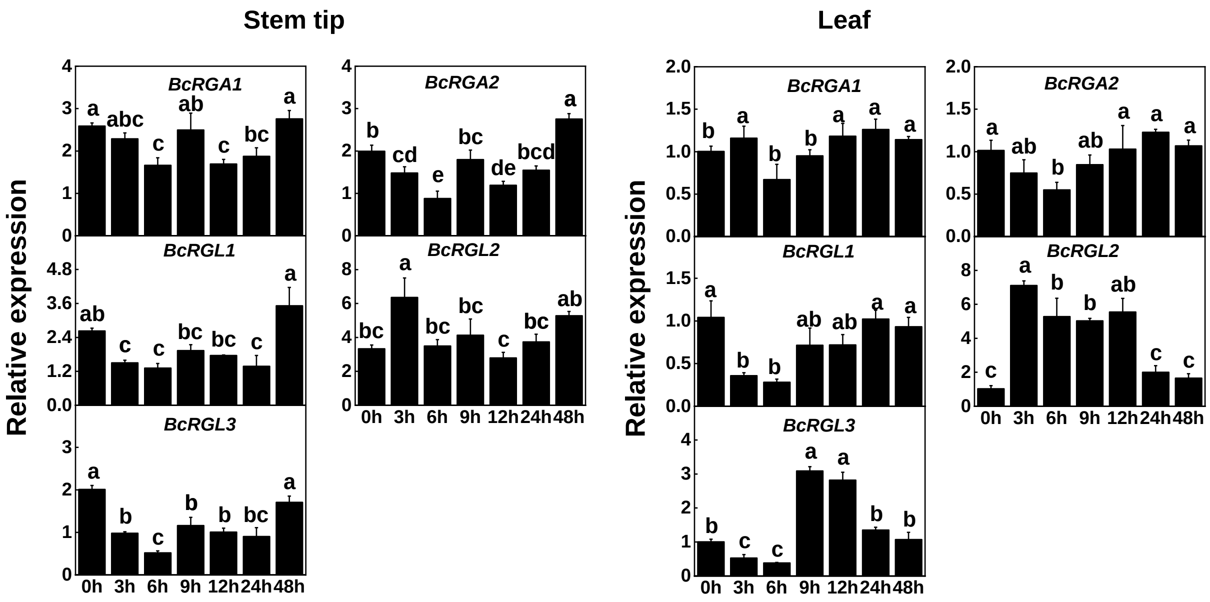

Figure S6. Expression profiles of flowering Chinese cabbage *DELLA* genes under three-true-leaf stage treatments at 15 °C. The data represent an average of three replicates ± standard error. Values followed by the same letter are not significantly different using Duncan's test at $p < 0.05$.

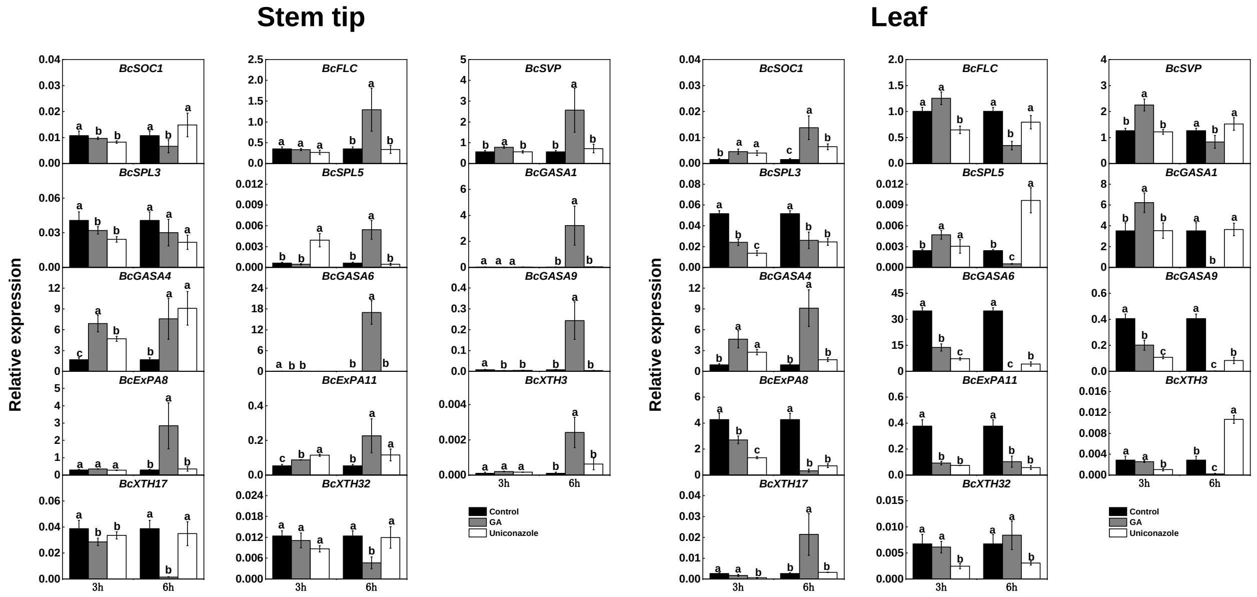

Figure S7. Expression profiles of flowering Chinese cabbage bolting and flowering-related genes under two-true-leaf stage treatments with gibberellin A3 (GA_3) and uniconazole. The data represent an average of three replicates ± standard error. Values followed by the same letter are not significantly different using Duncan's test at $p < 0.05$.

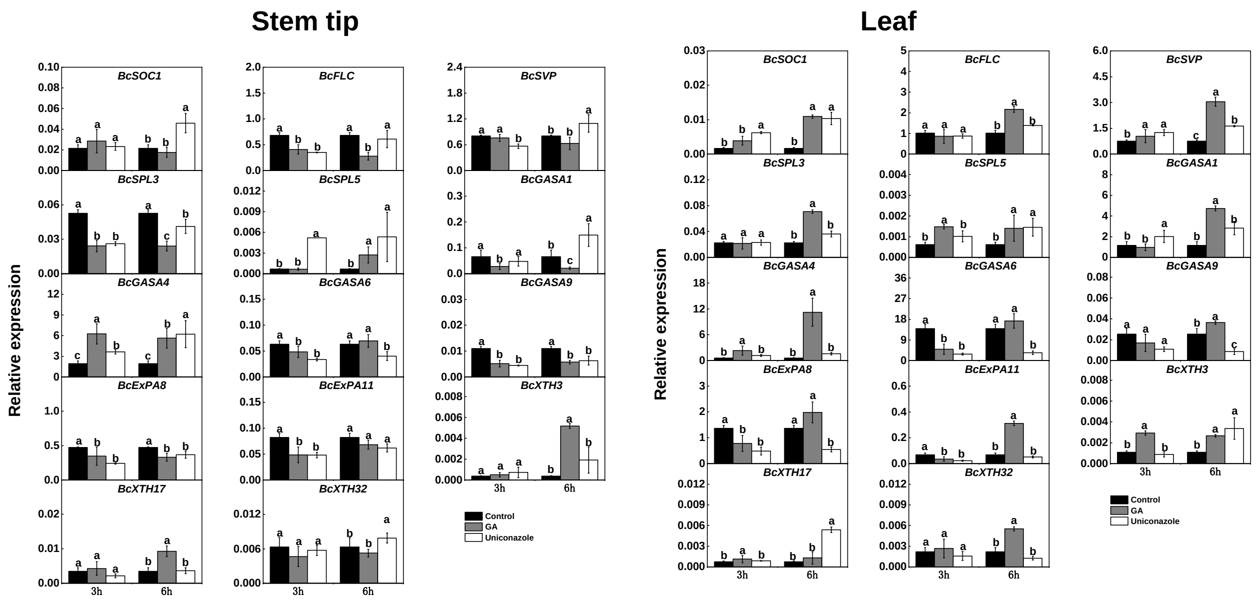

Figure S8. Expression profiles of flowering Chinese cabbage bolting and flowering-related genes under three-true-leaf stage treatments with gibberellin A3 (GA_3) and uniconazole. The data represent an average of three replicates ± standard error. Values followed by the same letter are not significantly different using Duncan's test at $p < 0.05$.

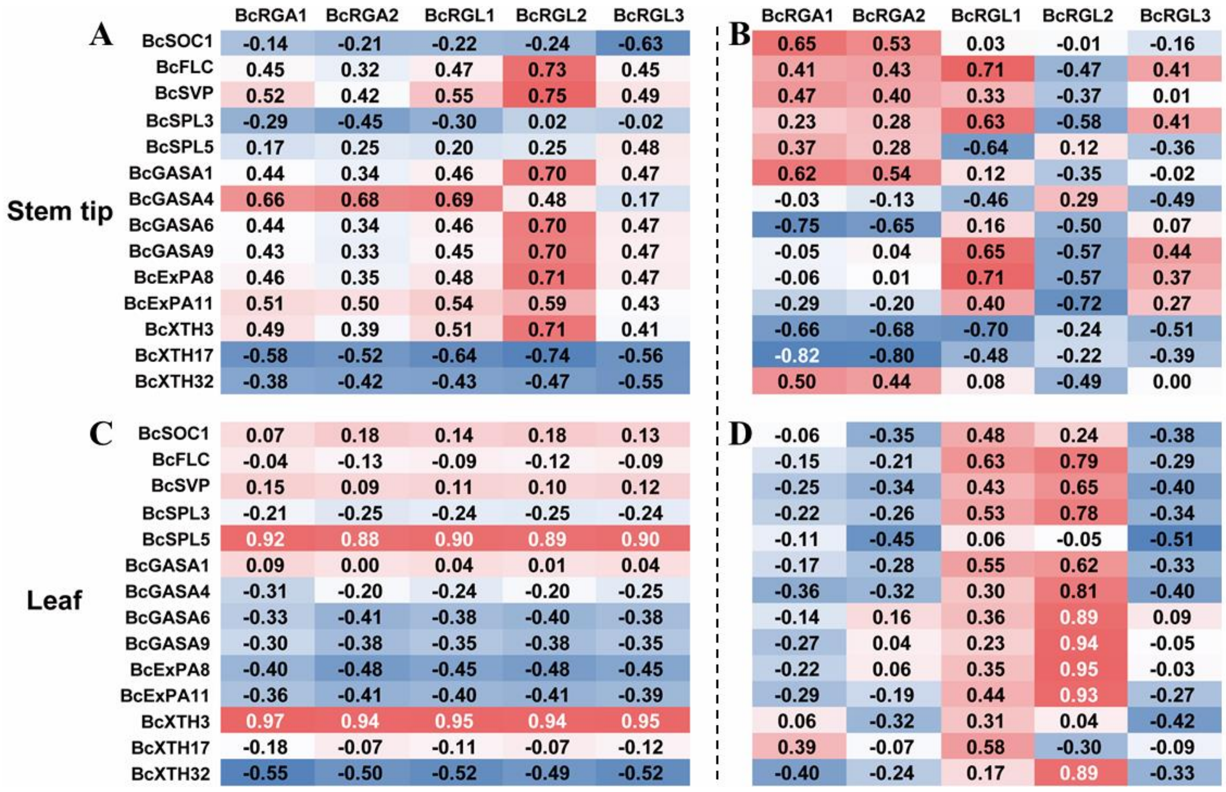

Figure S9. Correlation between gene expression patterns of *DELLA*, flowering, and elongation genes. Red: positively correlated; blue: negatively correlated. (**A**/**C**): two-true-leaf stage treatment, (**B**/**D**): three-true-leaf stage treatment. The white letters represent significant Pearson correlations (p<0.05).

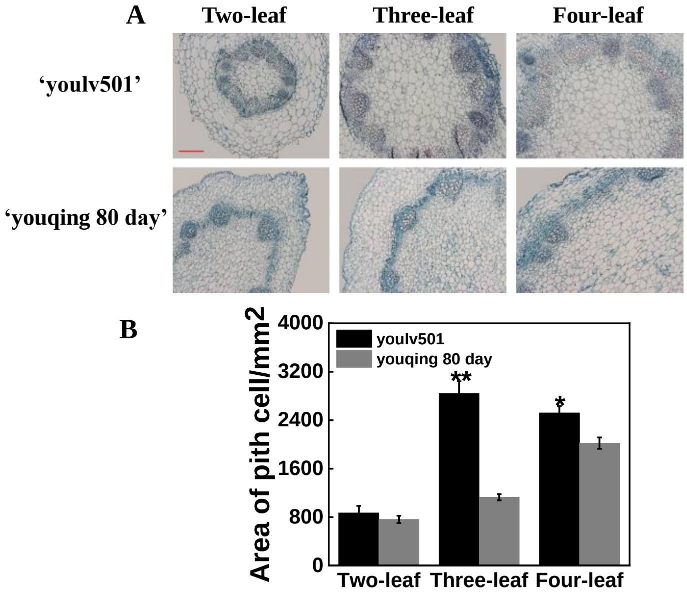

Figure S10. Stem tip horizontal structures of 'youlv501' and 'youqing 80 day' varieties of flowering Chinese cabbage. (**A**) Stem tip horizontal structures of three development stages. (**B**) Area of a pith cell in three development stages of flowering Chinese cabbage. All images were taken under a 10x microscope, and the scale was unified at 200 μm.
