# Supplementary figures and images for: Identification of DELLA Genes and Key Stage for GA Sensitivity in Bolting and Flowering of Flowering Chinese Cabbage
Source: Int J Mol Sci. 2021 Nov 9;22(22):12092. doi: 10.3390/ijms222212092 (PMC8624557; doi:10.3390/ijms222212092)

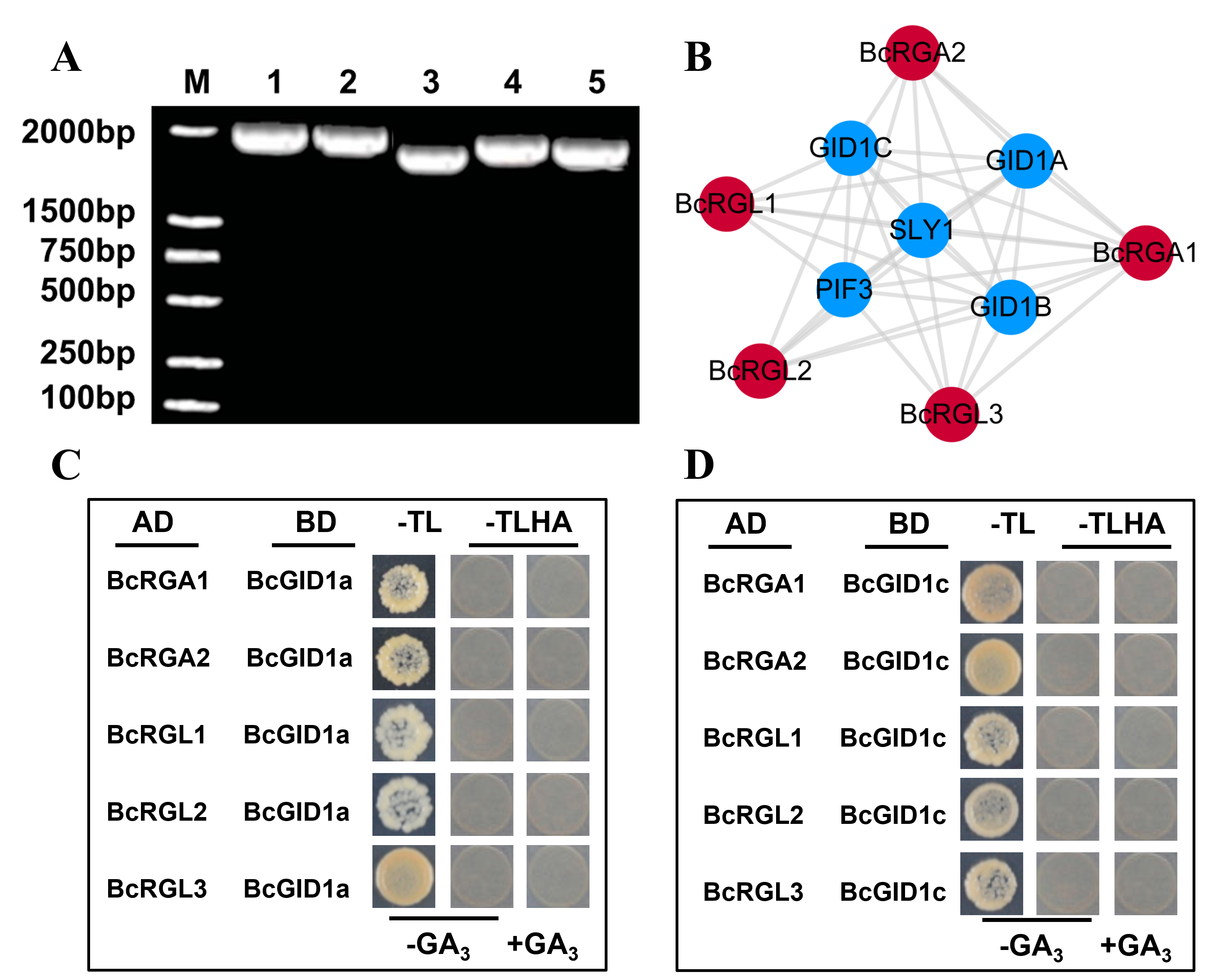

Supplement: Supplementary file 1 [file ijms-22-12092-s001.zip › supplementary figure-modified/Figure S1.tif]

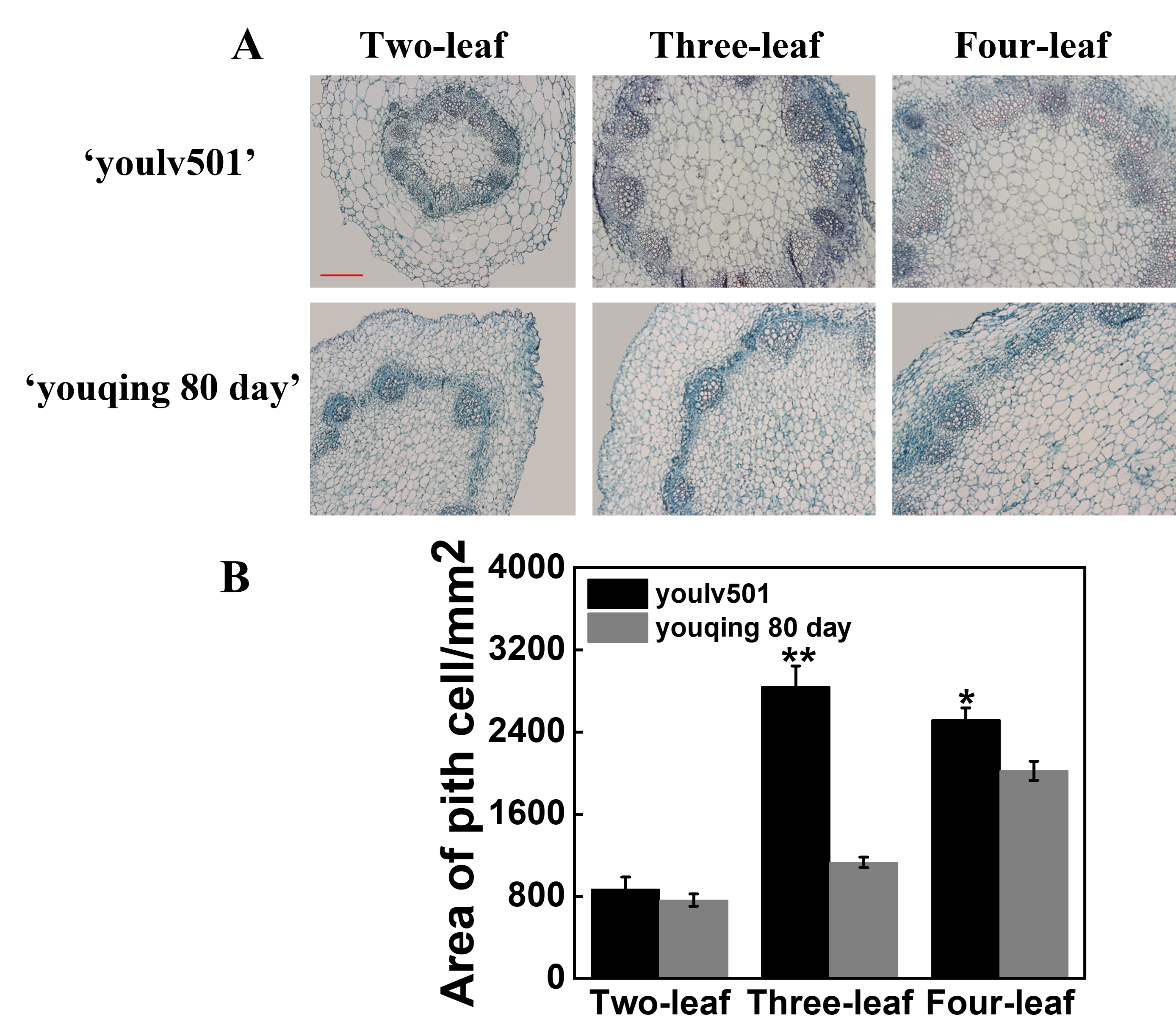

Supplement: Supplementary file 1 [file ijms-22-12092-s001.zip › supplementary figure-modified/Figure S10.tif]

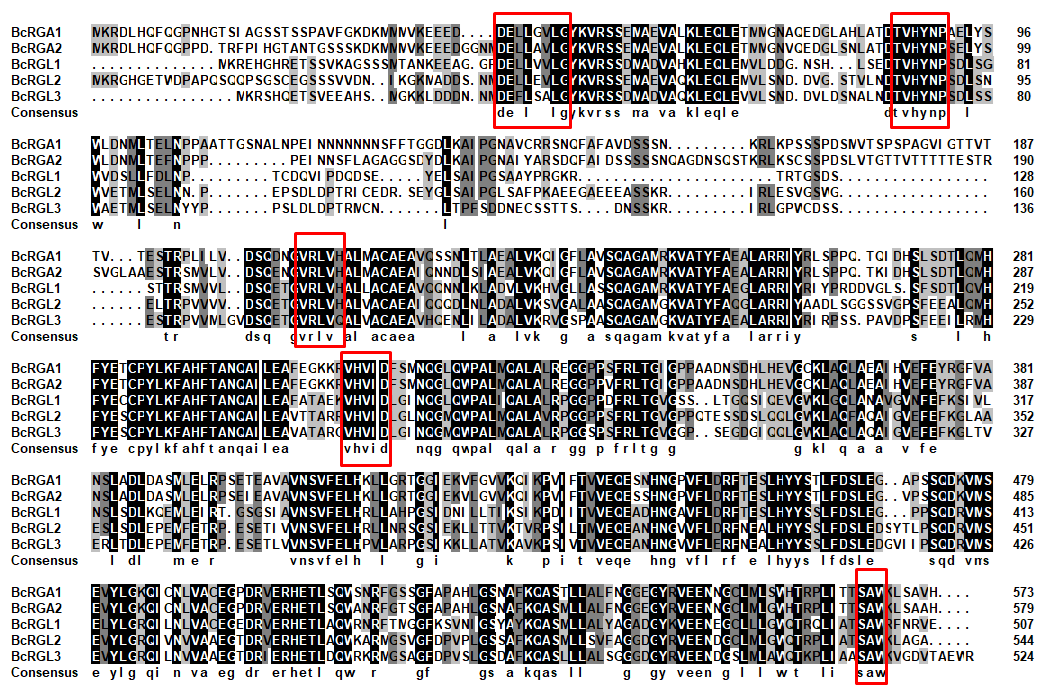

Supplement: Supplementary file 1 [file ijms-22-12092-s001.zip › supplementary figure-modified/Figure S2.tif]

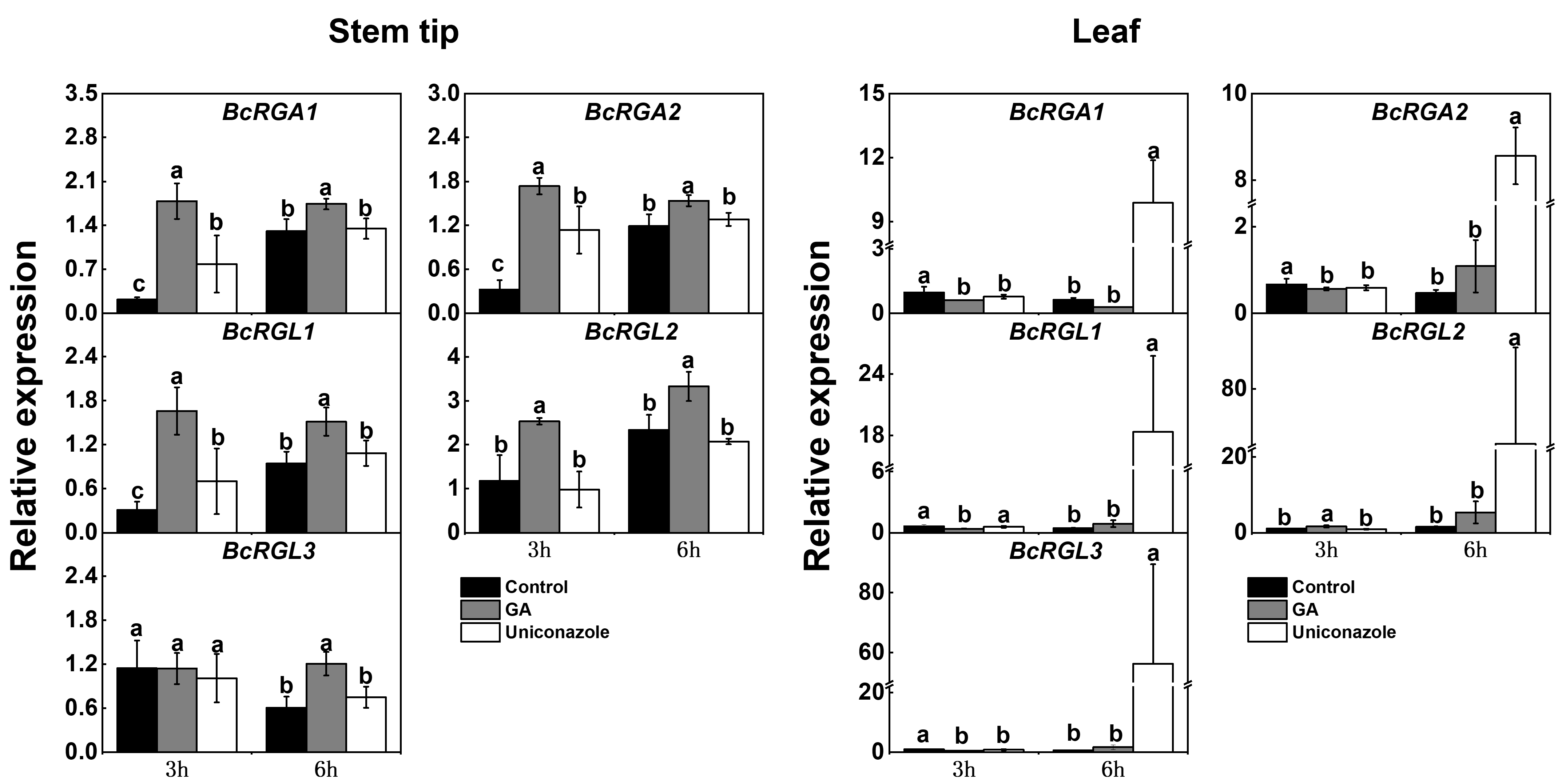

Supplement: Supplementary file 1 [file ijms-22-12092-s001.zip › supplementary figure-modified/Figure S4.tif]

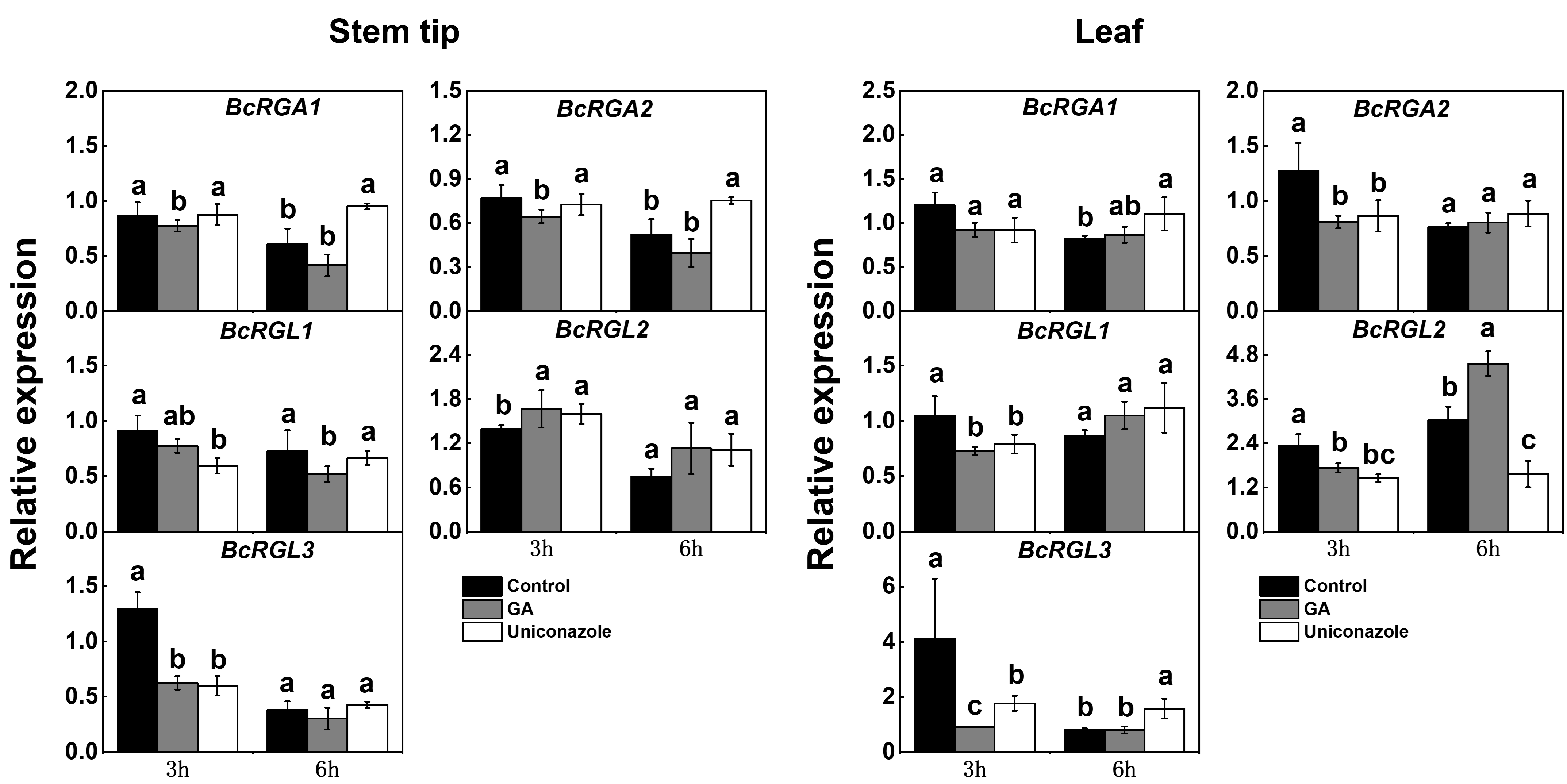

Supplement: Supplementary file 1 [file ijms-22-12092-s001.zip › supplementary figure-modified/Figure S5.tif]

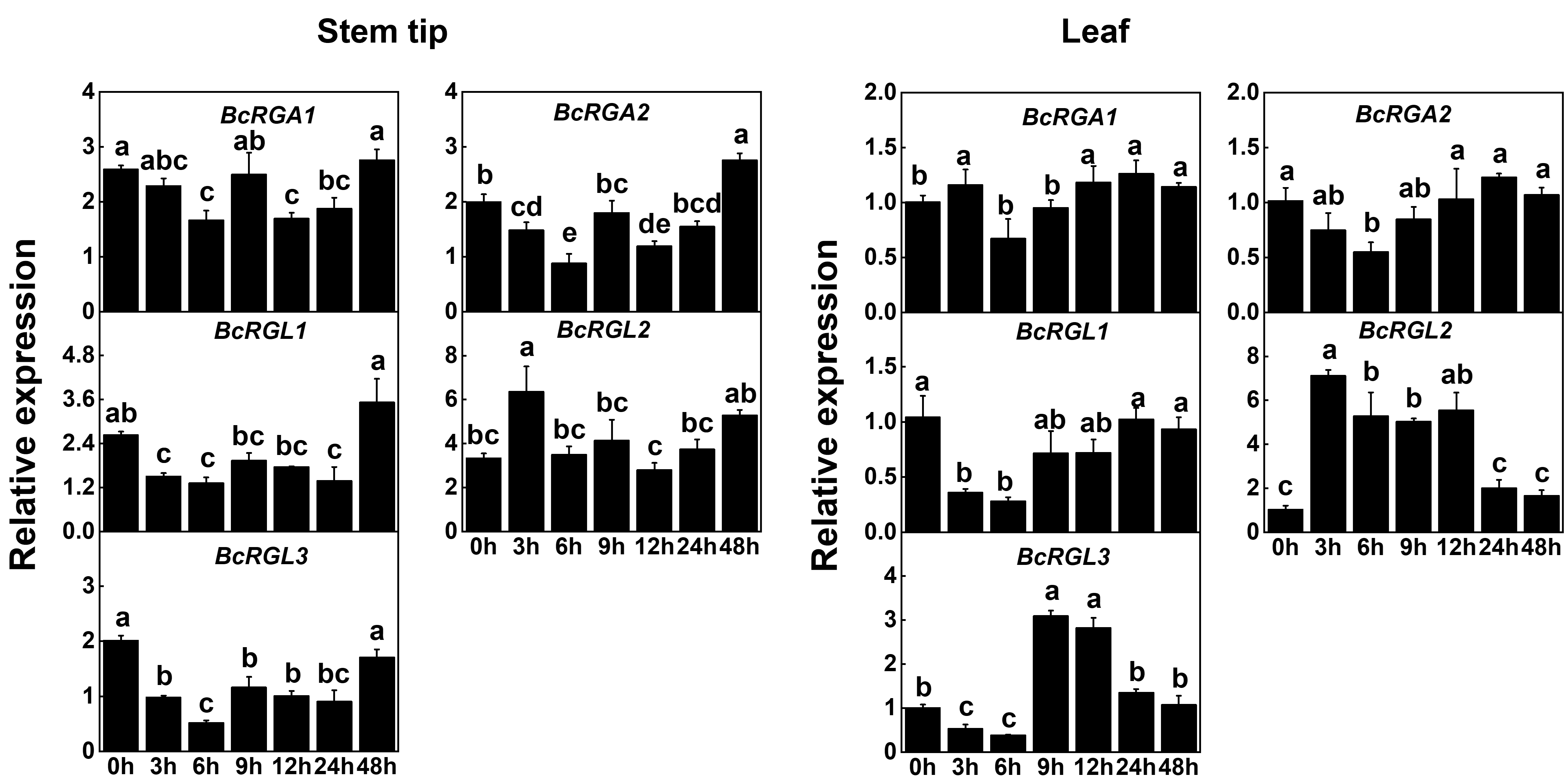

Supplement: Supplementary file 1 [file ijms-22-12092-s001.zip › supplementary figure-modified/Figure S6.tif]

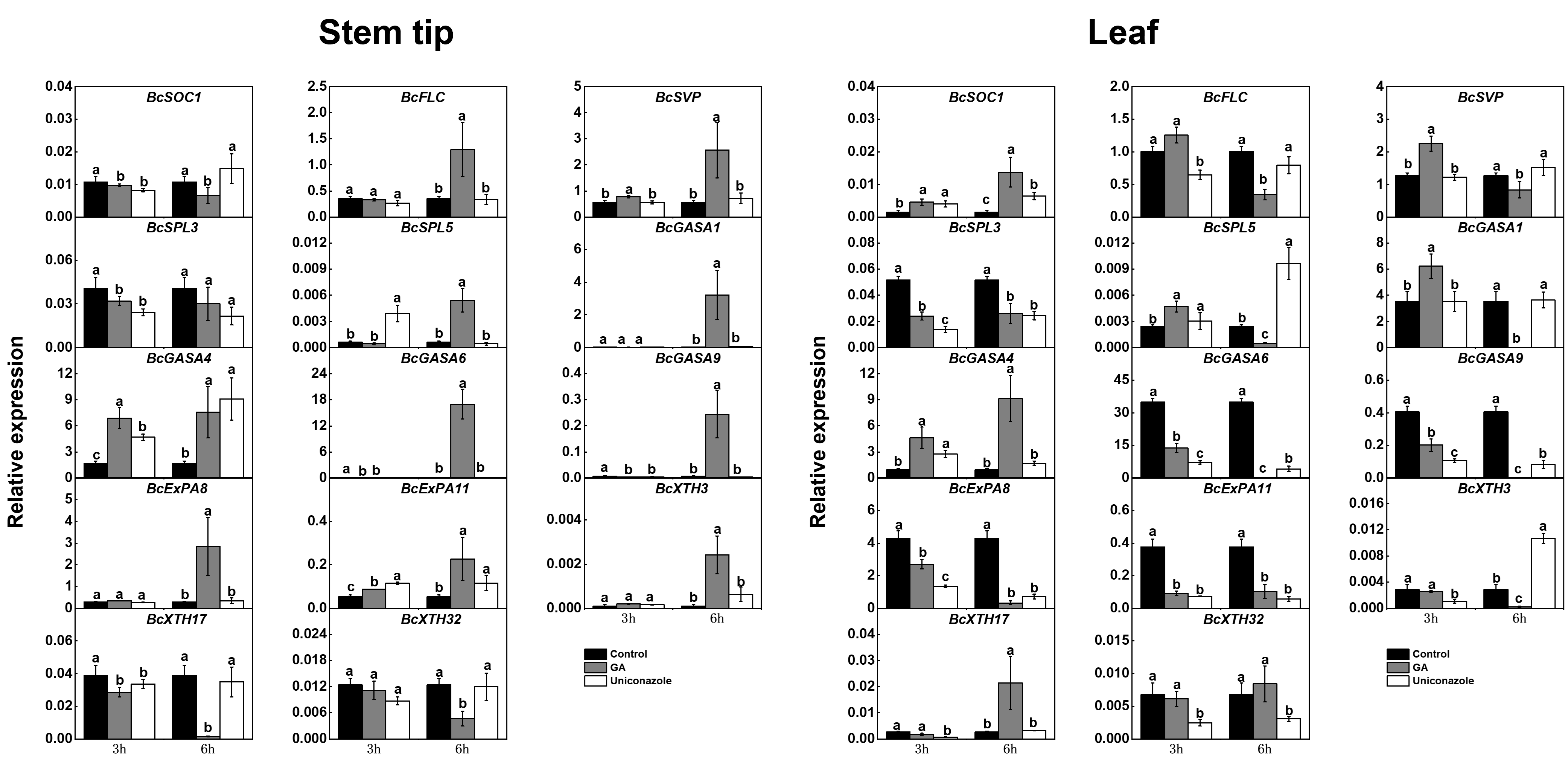

Supplement: Supplementary file 1 [file ijms-22-12092-s001.zip › supplementary figure-modified/Figure S7.tif]

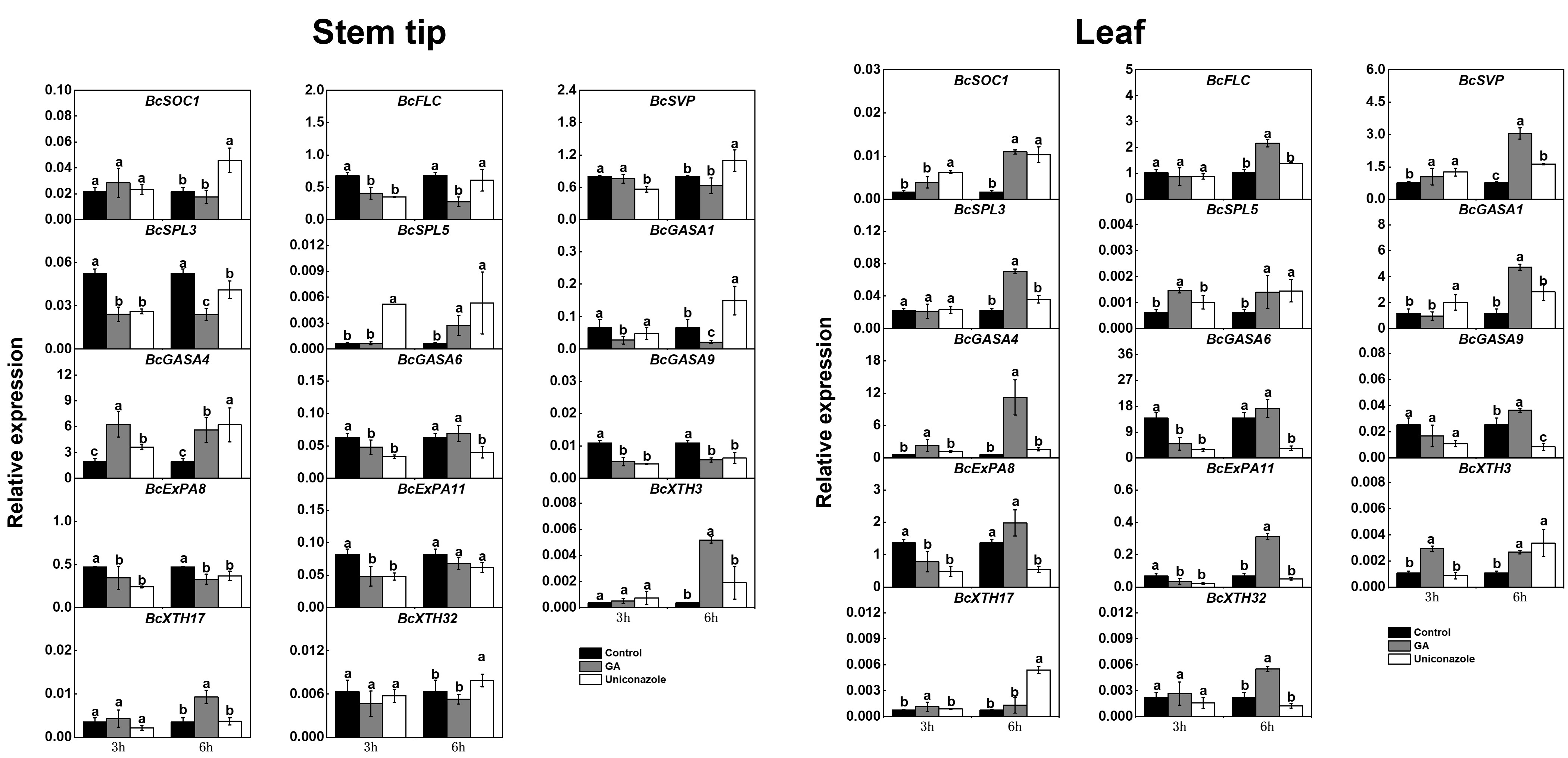

Supplement: Supplementary file 1 [file ijms-22-12092-s001.zip › supplementary figure-modified/Figure S8.tif]

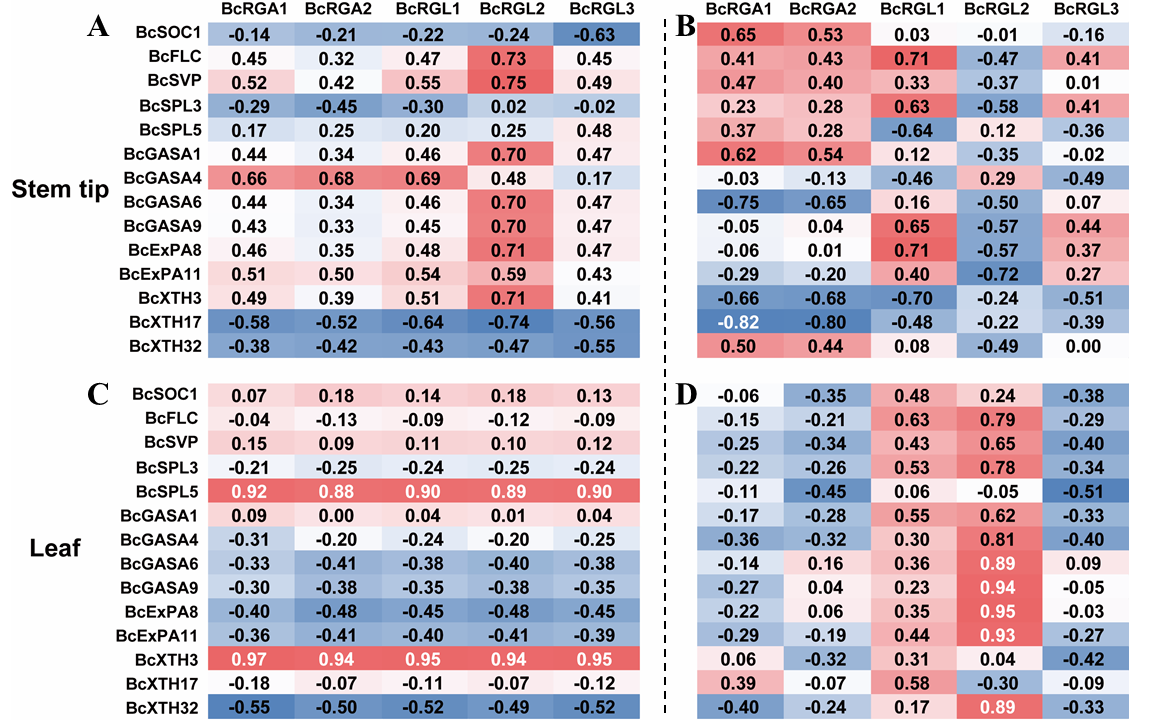

Supplement: Supplementary file 1 [file ijms-22-12092-s001.zip › supplementary figure-modified/Figure S9.tif]
